# The limestone spheroids of ‘Ubeidiya: intentional imposition of symmetric geometry by early hominins?

**DOI:** 10.1098/rsos.230671

**Published:** 2023-09-06

**Authors:** Antoine Muller, Deborah Barsky, Robert Sala-Ramos, Gonen Sharon, Stefania Titton, Josep-Maria Vergès, Leore Grosman

**Affiliations:** ^1^ Computational Archaeology Laboratory, Institute of Archaeology, Hebrew University of Jerusalem, Jerusalem, Israel; ^2^ Institut Català de Paleoecologia Humana i Evolució Social (IPHES-CERCA), Zona Educacional 4, Campus Sescelades URV (Edifici W3), 43007 Tarragona, Spain; ^3^ Departament d'Història i Història de l'Art, Universitat Rovira i Virgili, Avinguda de Catalunya 35, 43002 Tarragona, Spain; ^4^ MA Program in Galilee Studies, East Campus, Tel-Hai College, Upper Galilee, Israel

**Keywords:** spheroids, stone balls, Lower Palaeolithic, three-dimensional lithic analysis, spherical harmonics, ‘Ubeidiya‌

## Abstract

Spheroids are one of the least understood lithic items yet are one of the most enduring, spanning from the Oldowan to the Middle Palaeolithic. Why and how they were made remains highly debated. We seek to address whether spheroids represent unintentional by-products of percussive tasks or if they were intentionally knapped tools with specific manufacturing goals. We apply novel three-dimensional analysis methods, including spherical harmonics and surface curvature, to 150 limestone spheroids from ‘Ubeidiya (*ca* 1.4 Ma), presently the earliest Acheulean occurrence outside of Africa, to bring a new perspective to these enigmatic artefacts. We reconstruct the spheroid reduction sequence based on trends in their scar facets and geometry, finding that the spheroid makers at ‘Ubeidiya followed a premeditated reduction strategy. During their manufacture, the spheroids do not become smoother, but they become markedly more spherical. They approach an ideal sphere, a feat that likely required skilful knapping and a preconceived goal. Acheulean bifaces are currently thought to represent the earliest evidence of hominins imposing a premeditated, symmetrical shape on stone. The intentional production of sphere-like objects at ‘Ubeidiya similarly shows evidence of Acheulean hominins desiring and achieving intentional geometry and symmetry in stone.

## 1. Introduction

**Figure 1.**
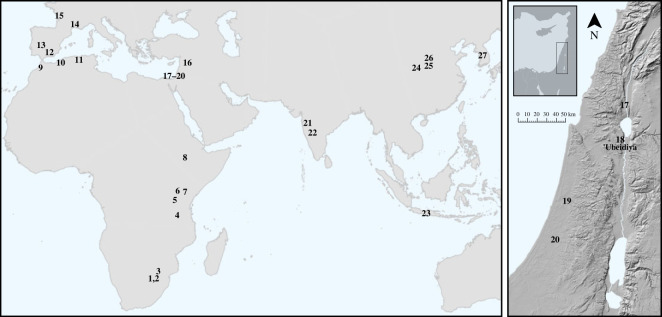
Left: A map of select sites where stone balls (including spheroids) are documented. Right: A map of sites with stone balls in the southern Levant, including the location of ‘Ubeidiya. 1. Sterkfontein [1]; 2. Kromdraai [2]; 3. Cave of Hearths [3]; 4. Isimila [3]; 5. Olduvai Gorge [4]; 6. Olorgesailie [5]; 7. Isenya [6]; 8. Melka Kunture [7]; 9. Thomas Quarry I [8]; 10. Ternifine [9]; 11. Ain Hanech, El Kherba and Ain Boucherit [10–13]; 12. Barranco León [14,15]; 13. Santa Ana [16]; 14. Bois-de-Riquet US4 [17]; 15. La Quina [18]; 16. Hummal [19]; 17. North of Bridge Acheulean locality of Gesher Benot Ya'akov [20]; 18. ‘Ubeidiya [21]; 19. Qesem Cave [22,23]; 20. Revadim [24]; 21. Chikri-on-Pravara [25]; 22. Hungsi Valley [26]; 23. Ngebung [27]; 24. Lantian [28]; 25. Sanmenxia [29]; 26. Fenhe River sites [30,31]; 27. South Korean river basin sites [32].

Spheroids have long puzzled archaeologists with their peculiar morphology and unclear functionality. What we do know is that they are among the most enduring lithic technologies, occurring in the Oldowan and the Acheulean, and even lasting into the Middle Stone Age/Middle Palaeolithic, and beyond. Geographically, spheroids appear to have originated in Oldowan and Early Acheulean toolkits in eastern Africa and are also found in southern, western and northern Africa. They are also documented sporadically in the Levant and Europe, and even as far as southern and eastern Asia (figure 1). See Willoughby [3] for a comprehensive summary of the history of research into spheroids, and Cabanès *et al*. [33] for a summary of their near-global spatio-temporal distribution.

Excavations at the Early Acheulean site of ‘Ubeidiya have yielded a large number of stone balls (electronic supplementary material, table S1), offering a unique opportunity to conduct quantitative and objective three-dimensional (3D) analyses to explore how and why these curious artefacts were made. ‘Ubeidiya is situated in the Dead Sea Rift Valley, at the northern margin of the Red Sea–East African Rift System. Dated to *ca* 1.4 Ma [34,35], the site presently constitutes the oldest evidence of the Acheulean outside of Africa. Its geographical position in the southern Levant makes it a key site for exploring the first Acheulean hominin forays out of Africa.

Such a large sample of spheroids is rare in the Lower Palaeolithic. At other Pleistocene sites in the Levant, stone balls (items typically called polyhedrons, sub-spheroids, spheroids and bolas) tend to occur only sporadically and in small numbers, if at all. Some examples from the Levant include 12 stone balls from the Oldowan layers at Hummal [19], 14 from Latamne [36], 3 from ‘Evron Landfill [37], 12 from the North of Bridge Acheulean locality of Gesher Benot Ya'aqov [20,38], 7 from Revadim [24,39] and 30 from the Amudian layers of Qesem Cave [22,23,39].

This paper reports new data about a large sample of 150 limestone spheroids from ‘Ubeidiya. Some view spheroids as existing within one long premeditated and well-organized reduction continuum; from the original nodule, to polyhedron, to sub-spheroid, to spheroid, to bola [40,41]. Others view them as unintentional by-products of other processes, such as expedient flaking [10,42], hammerstone use [43–45] or battering, pounding and crushing [46–48]. Our goal is to resolve this issue by way of objective 3D analyses aimed at reconstructing a plausible spheroid reduction sequence. If one exists, this analysis will enable us to hypothesize about the intentionality of spheroid manufacture and identify any goals of their production.

To do so, we compute a suite of 3D variables to characterize the complexity of spheroid geometry and explore whether they are best explained as percussors, cores or intentionally knapped items. These variables include scar attributes (edge angles and surface area), surface curvature and sphericalness.

Comprehensive 3D analysis methods have not yet been applied to spheroids. We introduce spherical harmonics to lithic shape analysis for the first time, creating a new metric for quantifying complex 3D shape variability of stone tools. The spheroids from ‘Ubeidiya provide a rare opportunity to explore these little understood artefacts and to address questions of hominin technological and cognitive evolution, such as the imposition of shape and symmetry on stone tools.

## 2. Methods

### 2.1. Archaeological context

The site of ‘Ubeidiya contains *ca* 60 distinct archaeological horizons bearing evidence for human occupation, estimated to represent a *ca* 100 000-year sequence of site occupation [49–52]. A series of geological trenches revealed four distinct members (in descending chronological order): Fu (fluviatile upper), Lu (limnic upper), Fi (fluviatile inferior) and Li (limnic inferior), each representing oscillating lake regressions and transgressions [52,53]. The Fi layers contain the majority of the archaeological finds, including most of the spheroids (with the remainder from Fu).

Thus far, ‘Ubeidiya has yielded a total of 598 flint, basalt and limestone stone balls from 38 distinct stratigraphic layers, most of which (57.5%) are flint polyhedron core forms [21]. Aside from stone balls, the remainder of the lithic assemblage comprises cores, flakes, choppers, assorted bifaces and trihedral tools. This assemblage has a distinct Early Acheulean character, most closely resembling the Upper Bed II sites of Olduvai Gorge [21,54].

### 2.2. Sample

A total of 150 limestone items were sampled from the complete stone ball assemblage described by Bar-Yosef & Goren-Inbar [21], as shown in electronic supplementary material, table S1. This sample is markedly larger than any previous spheroid assemblage studied using traditional methods of lithic analysis. All artefacts examined here are housed at the Institute of Archaeology, Hebrew University of Jerusalem (Mount Scopus Campus). All of our 3D models are made available in an open-access data repository [55].

Originally, Clark [56] defined stone balls as lithic artefacts of a spherical shape achieved via the knapping of facets. Later, Kleindienst [57] separated these stone balls into missiles (manuports or lightly knapped items used as projectiles), polyhedrons (artefacts almost completely covered in facets left by intersecting flake scars) and bolas (almost completely pecked and battered items leaving a smooth and spherical surface). Leakey [4] defined polyhedrons as angular forms displaying at least three worked edges, spheroids as items whose entire surface has been facetted or smoothed, and sub-spheroids as intermediate between these two types. Sahnouni *et al*. [10] further differentiated between facetted and battered spheroids.

An implicit assumption of these classification schemes is that each ‘type’ represents successive stages within one extended reduction sequence [40,41]. At ‘Ubeidiya, this continuum already appears insufficient, as differences in raw material and size clearly distinguish these categories. Bar-Yosef & Goren-Inbar [21] noted that spheroids were made on larger limestone nodules, while polyhedrons were smaller and made on flint. Similarly, at Olduvai Gorge (Beds I and II) and the Ounjougou complex, spheroids are larger than polyhedrons on average [58,59]. As knapping is a reductive process, wherein artefacts become smaller as a stone's volume is removed, the smaller polyhedrons at these sites are unlikely to represent an earlier phase of a spheroid reduction sequence.

As we are interested in better understanding spheroids, we analyse here only the limestone items from the stone ball assemblage, including those that are too angular to conform to traditional definitions of spheroids. Some would refer to these more angular limestone items as ‘polyhedrons’ and interpret them as earlier stages in the spheroid reduction sequence (e.g. [40,41]). However, at ‘Ubeidiya [21], the term ‘polyhedron’ has been used to refer to the smaller, mostly flint, cores and core-tools (a sub-type of ‘core-choppers’). One of the key findings of Bar-Yosef & Goren-Inbar's [21] lithic analysis was that the different raw materials at ‘Ubeidiya were used for distinct reduction sequences, making the flint stone balls entirely distinct from the limestone stone balls, which they classified as polyhedrons and sub-spheroids/spheroids, respectively. It is this latter group of artefacts that is the focus of this present study. However, the limestone component of the stone ball assemblage of ‘Ubeidiya does include items that some would call ‘polyhedrons’, meaning items that represent earlier stages of spheroid reduction. These two conceptualizations of ‘polyhedrons’ make their nomenclature confusing, so we will simply refer to the limestone component of the stone ball assemblage as ‘spheroids’.

Our sampling criteria were designed to capture the broadest possible morphology of limestone spheroids present at ‘Ubeidiya. We excluded broken items or any with concretions, as well as those with a ventral surface or any obvious sharp working edges. These criteria thus excluded any flakes and flake-tools, as well as items like choppers and bifaces. The example items displayed in figure 5 were chosen to represent the diversity of items in our sample, ranging from cubic and angular to remarkably spherical. Electronic supplementary material, table S1, shows the layers from which we sampled and the counts from each layer. Again, we aimed for a diverse sample, including items from as many of the layers that contained limestone spheroids as possible. A comparison of the spheroids from different layers is beyond the scope of this present study. We treated the spheroid assemblage as one sample, as previous lithic analyses at ‘Ubeidiya demonstrated that the major intra-site variability related to the frequency of different tools in the different layers, rather than in the techno-morphology of the tools themselves [21,54].

### 2.3. Three-dimensional scanning

The spheroids were scanned using a Polymetric PT-M structured light scanner (depth resolution: 6 µm). These high-resolution 3D models were processed with the *Artifact3-D* software [60–62]. *Artifact3-D* [62] was developed at the Computational Archaeology Laboratory of the Hebrew University of Jerusalem. The open-source code for both *Artifact3-D* and the spherical harmonic functions is available for download from https://sourceforge.net/projects/artifact3-d/.

### 2.4. Edge angle analysis

**Figure 2.**
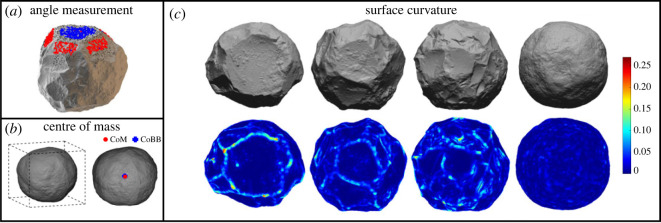
Methods of measuring edge angles, centre of mass and surface curvature. (*a*) The method of calculating angles on the spheroids, using the Angle Between Surfaces function of the Artifact3-D program. The red and blue dots represent coordinates comprising the angle calculation on two different surfaces. (*b*) An example spheroid's minimum bounding box (dashed lines) as well as the location of its centre of mass (CoM; red circle) and centre of bounding box (CoBB; blue cross). (*c*) Surface curvature values shown with heatmaps of example spheroids. Regions of higher curvature are shown in brighter colours.

After processing, *Artifact3-D* was used to precisely calculate angles on the surface of the spheroids (figure 2*a*) following the procedure outlined by Valletta *et al*. [63]. Two surfaces were demarcated by selecting a number of points at the intersection of the surfaces. The *Angle Between Surfaces* function then compares the mean angle between 3D coordinates (red and blue dots) on the most regular portion of these two surfaces. These edge angle values were calculated to explore the variability of the spheroid surface. In general, the more spherical an object, the higher the angles on its surface. This also allows a quantification of the relationship between particular scars and the remainder of the spheroid surface.

### 2.5. Centre of mass

*Artifact3-D* was also used to compute the centre of mass (CoM) and the centre of the bounding box (CoBB) (figure 2*b*). In 3D geometry, the bounding box is the smallest rectangular prism that encloses an object in a given orientation. It circumscribes an object according to its maximum orthogonal measurements. The CoBB is thus at the mid-way point of the maximum length (*y*-axis), width (*x*-axis) and thickness (*z*-axis) dimensions of each of the artefacts. Meanwhile, the CoM is calculated by multiplying the surface area of each triangular face of the mesh by the vector of each of these triangles, then normalizing according to the total surface area. *Artifact3-D* automatically positions the CoM of each artefact at *xyz*-coordinates of 0, 0, 0. This value represents the volumetric centre of the object. For objects made on homogeneous material, this volumetric centre thus approximates the CoM.

The proximity between the CoM and CoBB (normalized by the artefact's volume) reveals how evenly an object's volume is distributed. The CoM is derived from an object's entire volume and is thus relatively insensitive to surficial variations. On the other hand, even subtle protuberances on an object's surface will expand its bounding box and shift the CoBB away from the CoM. A perfect sphere will possess identical CoM and CoBB values. As the artefacts become more spherical, their CoM and CoBB values should converge.

### 2.6. Surface curvature

To quantify the roughness of each artefact's surface we compute the surface curvature at each face of the 3D mesh. In two and three dimensions, respectively, curvature is simply the extent to which a curve differs from a straight line, or a surface differs from a plane. We compute this 3D curvature iteratively on a very small scale over the entire object's surface, and then take the average of these curvature values for a quantification of overall surface roughness.

Specifically, at each face of the 3D mesh, its closest faces (using the k-nearest neighbours algorithm) were used to approximate a plane. The k-nearest neighbours algorithm identifies the closest 3D coordinates to a given coordinate in Euclidean space. To normalize for artefact size and scan resolution, each cluster of k-nearest neighbours was set at 0.05% of the total surface area. On our actual spheroids, with non-flat surfaces comprised of flake scars, facets and cortex, the deviation from this local plane quantifies curvature at a local scale. Surface roughness is computed by averaging every local curvature value from each face of the 3D mesh (figure 2*c*). Flake scar margins, facets, pecking marks and rough areas of cortex all contribute to high curvature values, whereas the interior of flake scars and smooth/rounded areas possess lower curvature values.

### 2.7. Spherical harmonics

Spherical harmonic functions can be used to approximate 3D shapes like how Fourier transforms approximate curves in Cartesian space. They are perhaps best known as the eigenfunctions of orbital angular momentum that occur in a great variety of physical problems such as gravity, hydrodynamics and mechanics.

The spheroids are positioned by the normal vectors that calculate the surface tensor whose eigenvectors reflect three orthogonal planes. Then, we follow the method that applies the spherical harmonics transform to three independent spherical functions representing the spherical parametrization of the local coordinates of the mesh *x*, *y* and *z* [64]. Their shape is transformed as functions on the unit sphere by describing each surface point by its spherical coordinates (*r*, *θ* and *φ*), where *r* is the distance from (0, 0), *θ* is the latitude measured from the *z*-axis and *φ* is the longitude measured in the *xy* plane from the *x*-axis to *y*-axis. We describe the shape function by the coefficients of a real spherical harmonics expansion. Since any function on the unit sphere can be expanded in this manner, the method uses the coefficients of the spherical harmonics expansion to describe the deformation parameters of each spheroid. Shape similarity is computed as the distance in coefficient space from the origin. These coefficients are unique and can thus be used as a vector for describing the shape.

**Figure 3.**
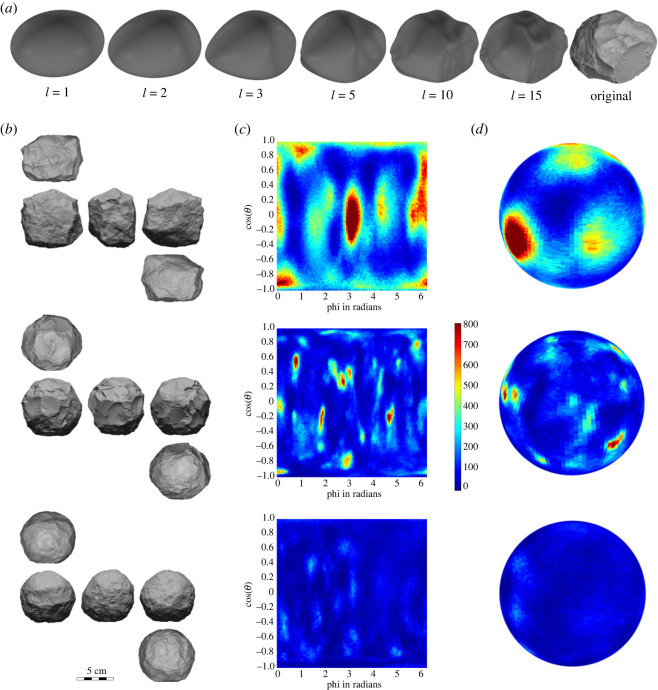
Spherical harmonics methods. (*a*) Spherical harmonic reconstruction of an example spheroid, with increasing numbers of coefficients (harmonic order *l*). (*b*) Example spheroids. (*c*) Two-dimensional and (*d*) 3D density matrices of Gauss maps for each of the example spheroids of the same row showing the extent of deviation from a spherical form. Brighter colours represent areas of less sphericalness, showing both the overall sphericalness as well as the location on each artefact where its shape deviates most from a sphere.

Beginning with a sphere, a spherical harmonic expansion with an increasing number of coefficients can approximate an object's 3D morphology, with more coefficients resulting in a more precise 3D reconstruction (figure 3*a*). The first three coefficients model how closely an object adheres to a perfectly spherical shape. Additional coefficients relate more to the surface topography and scars or facets of the spheroid, rather than to its underlying adherence to a spherical morphology. For a perfect sphere, the value of each of the first three coefficients is 1/3. The sum of the absolute difference between each coefficient and this ideal value offers a quantification of the extent to which an object deviates from a perfect sphere (sphericalness). The more these coefficients vary, the more the artefact deviates from a sphere. Here we use the term ‘sphericalness’ instead of ‘sphericity’ as the latter already possesses a mathematical definition, i.e. the ratio of the surface area of a sphere of equal volume as an object to that object's actual surface area. As shown in the Gauss maps (figure 3*b*,*c*), the colour of each cell (100 × 100) denotes the amount of deviation from a perfect sphere at each spherical coordinate. This allows a quantitative exploration of the areas of each artefact that most deviate from a sphere.

## 3. Results

### 3.1. Trends in spheroid reduction

**Figure 4.**
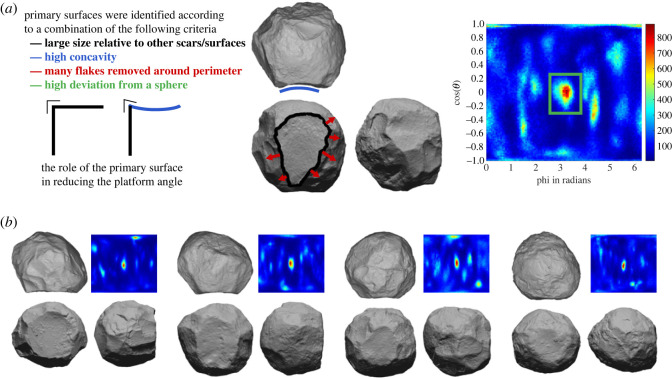
Primary surfaces of spheroids. (*a*) The criteria (colour-coded) from which primary surfaces were identified. Note the large centrally located scar (black), possessing high surface concavity (blue), serving as the platform for many flake removals (red) and significantly deviating from a sphere (green) in the Gauss map. The importance of primary surface concavity in reducing the external platform angle for subsequent flaking is also shown. (*b*) Further examples of spheroids with primary surfaces, showing the tendency for this primary surface (centre of the plots) to be the region of the artefact most distant from a spherical form.

The 150 limestone artefacts examined here either conform to traditional definitions of spheroids, or are too angular to be typically considered spheroids but could foreseeably be further knapped into spheroids. Starting with spheroids and working backwards, regardless of whether they were intentionally knapped or not, we know that they were ‘reduced’ into this spherical shape. Thus, we seek any trends in the scar and shape parameters of the 150 items. Most notably, a visual and quantitative analysis of these spheroids revealed that each one possessed a reserved ‘primary surface’, from which much of the reduction sequence proceeded. These primary surfaces are usually a large flake scar removed very early in the sequence, or occasionally a flat plane, likely corresponding to breakage during a cobble-opening phase, or a slightly concave cortical/weathered surface. These surfaces are identifiable according to the criteria outlined in figure 4.

**Figure 5.**
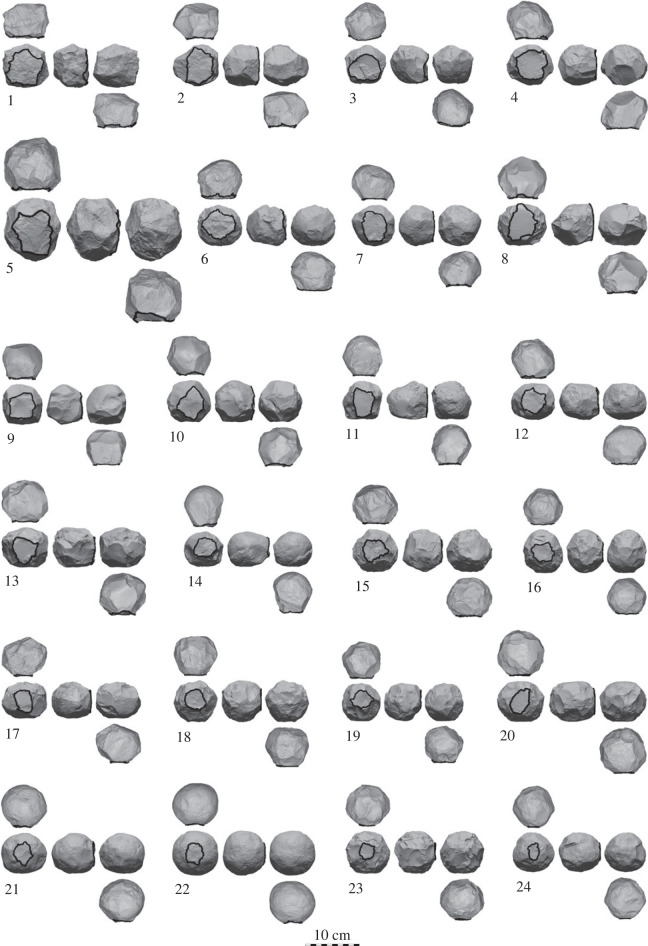
Twenty-four example artefacts belonging to the spheroid reduction sequence, showing the tendency for the primary surface to become smaller as the items become more spheroid-like.

We sought any trends in spheroid morphology to reconstruct a possible reduction sequence for these 150 items. To visualize these trends, the 24 example spheroids in figure 5 are ordered according to the primary surface's proportion of total surface area (descending). The surface area is precisely calculated (mm^2^) in *Artifact3-D* using the scar segmentation analysis [62,65]. The items are manually oriented according to this primary surface, and the primary surfaces are outlined in black.

**Figure 6.**
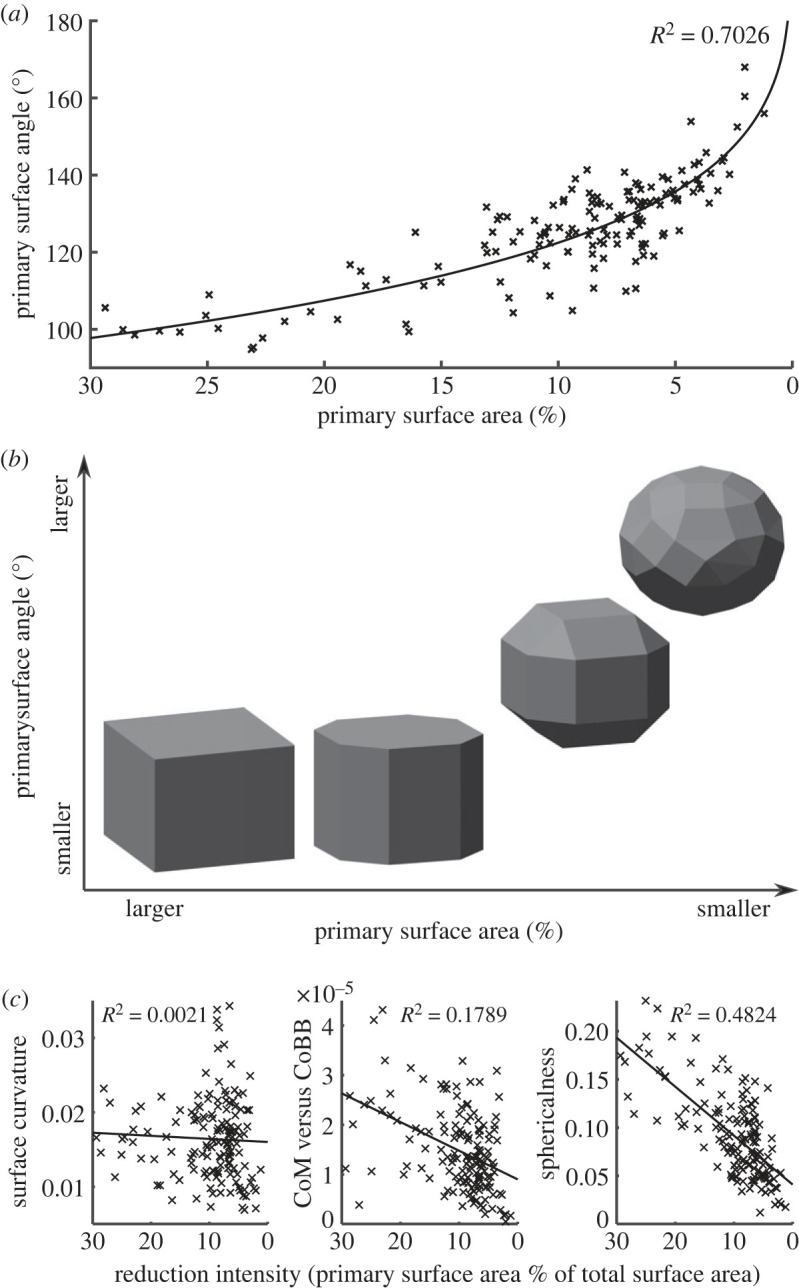
The proposed spheroid reduction sequence and its influence on key artefact variables. (*a*) The relationship between the size of the primary surface (as a proportion of total surface area) and the angle between the primary surface and the remainder of the object. Note the inverted *x*-axis. As the primary surfaces get smaller, the primary surface angle gets larger. (*b*) A model of the geometry of the proposed reduction sequence, showing how the flaking strategy affects spheroid morphology. Flaking around the perimeter of the primary surface reduces its surface area. More advanced flaking further reduces this surface area as well as increases the primary surface angle. (*c*) Scatter plots of the size of the primary surface versus various measures of the artefacts' morphology and surface characteristics. Surface curvature is uncorrelated with reduction intensity, while the latter two plots show that the spheroids become markedly more spherical as reduction proceeds.

Even from this visual comparison, it appears that as the primary surface becomes smaller, the angle between this primary surface and the remainder of the artefact increases, and the overall geometry of the artefacts become more spheroid-like. These trends are quantified in figure 6*a*, showing the relationship between shrinking primary surface area and increasing edge angle. Figure 6*b* summarizes this process with geometric approximations. The primary surface gets smaller the more it is used as a platform to remove flakes from around its perimeter. As such, the surface area of the primary surface (as a proportion of the total surface area) provides a useful proxy metric for reduction intensity and can be used to explore further trends in the reduction process.

Figure 6*c* shows how the 3D geometry and surface of the spheroids change as reduction proceeds (i.e. as the primary surface becomes smaller). Interestingly, there is almost no relationship between surface curvature and reduction intensity (*R*^2^ = 0.0021). Although some pieces appear to be rounded either via knapping/crushing/pecking or via weathering, other heavily reduced pieces possess high surface curvature values owing to the high number of facets remaining on their surface. Surface smoothness or roundedness, therefore, does not appear to be a goal of this reduction process. Assaf & Baena [39] experimentally found that repeated percussive use relatively quickly results in spheroids with smoother surfaces, similarly showing that smoothness was likely not intentional.

Instead of a surficial measurement like curvature, a volumetric measure like the comparison between the CoM and the CoBB reveals modest improvement in the evenness of mass distribution throughout the reduction sequence (*R*^2^ = 0.18). The spherical harmonic analysis reveals, not just the evenness of mass distribution, but also how closely it conforms to a sphere. This analysis revealed that reduction intensity is most strongly correlated with sphericalness (*R*^2^ = 0.48). During reduction, the pieces do not become smoother, but they become markedly more spherical.

### 3.2. Proposed reduction sequence

We propose the following reduction sequence based on the trends in scar attributes shown above. Knapping appears to commence by establishing a primary surface by either selecting a natural flat/concave surface on a cobble, creating a surface via breakage or by removing a large flake. Next, reduction appears to proceed via exploiting this primary surface by removing flakes around its perimeter.

As shown in figure 4*a*, this primary surface is used as a platform to remove many of the subsequent flakes. When needed, bidirectional flaking is undertaken, using the surface opposing the primary surface as a platform. These first steps are similar to the reduction sequence identified for the sub-spheroids at the Iberian Oldowan site of Barranco León [15].

After flaking around the perimeter of the primary surface, the intersection between the primary surface and the remainder of the spheroid is still at approximately a right angle. These sharper angles were removed, either via direct, passive or bipolar percussion, or using other actions, such as abrasion, crushing or pounding. Experiments are underway to test how these methods can create a spherical artefact. Regardless of the specific technology used, this phase of the reduction sequence led to an increase in the angle between the primary surface and the remainder of the object, as well as a decrease in the amount of primary surface left intact (figure 6*a*). As the angle increases and the primary surface area decreases, the object becomes more spherical (figure 6*c*).

The earliest stage of this proposed reduction sequence that has been reconstructed here involves establishing or exploiting a primary surface. At this stage, the items can be angular or even cubic-shaped. From this stage, there appears to be a continuous reduction sequence that ends with almost perfectly spherical spheroids. Thus, our findings conform most with those who argued that spheroids (or even so-called bolas) represent the final stage in a reduction sequence (e.g. [40,41]). The typological distinctions between polyhedrons, sub-spheroids and spheroids that are made in traditional definitions of these artefacts [4,57] may thus need rethinking. For most lithic artefacts, those belonging to early parts of the same reduction sequence are not typically classified as different types. We suggest that all limestone items examined here that fit within the proposed spheroid reduction sequence could be more simply called spheroids, even those that are more angular or even cubic.

It should be noted that this reduction sequence may not necessarily occur in one uninterrupted sequence, like at Qesem Cave where it has been argued that the spheroids were recycled after a long hiatus [22,23,39]. Evidence of recycling, like patination, different wear patterns, or scar hierarchies, is not readily visible on the spheroids of ‘Ubeidiya, at least partly due to the limestone raw material.

This 3D analysis also hints at how knappers overcame the physical practicalities of making a spherical object out of stone. All artefacts within the spheroid reduction sequence possessed a primary surface, typically a large flake scar removed early in the sequence, from which a high proportion of the subsequent flakes were removed. Much like platform rejuvenating flakes or tablets in typical core reduction, the concavity of this primary flake scar provides the knapper with a platform surface with a slightly but crucially lower platform angle (figure 4*a*). This may help explain how knappers were able to achieve spherical objects despite the physical limitations of striking high-angled platforms. Much of the early shaping could likely have been achieved using this strategy. However, we suspect that either bipolar-on-anvil or passive percussion would be necessary to remove the ridges in the latter parts of the reduction sequence and achieve the final facetted or smooth spherical form.

## 4. Discussion

We measured scar attributes, surface smoothness and sphericalness to assess the three most prevalent explanations of spheroids: percussor [43–48], core [10,42] or intentionally knapped item [40,41]. If spheroids represent hammerstones or percussors, then they should become both smoother and more spherical, as demonstrated by the experiments of Schick & Toth [44,45]. If spheroids represent expedient cores, then we expect to see some pattern in the removal of scars. This pattern could take the form of scars of regular size, orientation or distribution over the surface. The spheroids may even become smoother during a final stage of battering attempts to remove flakes. However, we do not expect them to become more spherical. While even random flaking can mimic intentional artefact shaping [66], this strategy is unlikely to approach anything as unnatural as a true sphere. Due to the limits on the fracture mechanics of flaking that govern detachable platform angles, the high-angled flake removals necessitated by a spherical form would require much more effort and shaping than is involved in expedient flaking.

Lastly, if spheroids represent intentionally knapped items, then we should see some pattern in the size, orientation or distribution of scars, and they should become more spherical, but not necessarily more rounded or smoother. Surface smoothness can be the result of natural phenomena, while sphericalness, where an item's geometry approaches that of a true sphere, is very rare in nature. For instance, river cobbles can become smoother with more rounded edges after long periods submerged in running water, but they almost never approach a truly spherical shape [67]. Support for human intentionality in the shaping of spheroids necessitates evidence of a repeated pattern in the scar distribution on the spheroids, and confirmation that a spherical form was the intended goal. If this is the case, the more reduced spheroids should possess a more spherical shape.

Our results conform most closely to the last explanation, that spheroids represent intentionally knapped items. A clear pattern in scar attributes was observed, whereby a primary surface was used as a platform to remove further scars. This primary surface became smaller and higher angled as reduction proceeded. The intentionality of these scar removals is supported by the experimental findings of Assaf & Baena [39], who found no macro flake removals occurred during repeated percussive use aimed at marrow extraction. Moreover, the spherical harmonic analysis revealed that reduction also resulted in the spheroids becoming more spherical. This procedure compares each artefact's morphology with that of an ideal sphere. It is sensitive to even subtle, imperceptible deviations from this ideal. Since spheres occur uncommonly in nature, it appears that this high level of sphericalness observed among the more reduced spheroids is likely to have been produced intentionally. As surface curvature was uncorrelated with reduction intensity, the knappers at ‘Ubeidiya did not desire a smooth, approximately round object. Nor did they accidentally produce a smooth surface via intensive percussion. Instead, it appears that they attempted to achieve the Platonic ideal of a sphere and they approached this ideal following their spheroid reduction sequence.

This imposition of a spherical form on knapped material has important evolutionary and cognitive implications. What sets Acheulean bifaces apart from earlier technologies like Oldowan choppers is their deviation from the original nodule form. Choppers require only a few flake removals and the final artefact form often resembles the original nodule shape. The significance of handaxe symmetry has been much discussed [68,69] especially regarding the likely need for a preconceived goal [70]. Acheulean bifaces like cleavers and handaxes represent early evidence of hominins imposing a geometric shape and symmetry on their stone tools. If symmetry was desired by knappers of Acheulean handaxes, which are symmetrical in three axes, then spheroids, symmetrical in all directions, also fulfil this need. Thus, spheroids also likely required similar levels of skill, forethought and planning to those encountered in handaxe manufacture [69,71–75].

Aside from the remarkable emphasis on achieving a spherical shape, the most notable feature of spheroids is perhaps their longevity and widespread geographical distribution. Occurring from the Oldowan to at least the Middle Palaeolithic, they represent one of the longest lasting lithic technologies, both preceding and outlasting even handaxes. The presence of spheroids prior to the onset of the Acheulean [13,15,48] may make our findings from the Acheulean of ‘Ubeidiya relevant to the Oldowan also. Our results suggest that the spheroids of ‘Ubeidiya are a complex formal technology that represent a manifestation of the complex cognitive and skilful capacities of Early Acheulean hominins. If similar intentional shaping can be demonstrated on Oldowan spheroids, this would likely represent the earliest evidence of hominins imposing a desired symmetrical geometry on their tools.

## Data Availability

The software and open-source code for the Artifact3-D program and the spherical harmonics analysis can be found in perpetuity at the following link: https://sourceforge.net/projects/artifact3-d/. This program is available with the open-source MIT licence. Future updates to the program will be deposited there, but the current version will remain available indefinitely also. All 3D models used in this analysis have been deposited in the Dryad Digital Repository: https://doi.org/10.5061/dryad.9ghx3ffp3 [55]. The data are provided in the electronic supplementary material [76].
